# Large language models predict human sensory judgments across six modalities

**DOI:** 10.1038/s41598-024-72071-1

**Published:** 2024-09-13

**Authors:** Raja Marjieh, Ilia Sucholutsky, Pol van Rijn, Nori Jacoby, Thomas L. Griffiths

**Affiliations:** 1 Department of Psychology, Princeton University, Princeton, USA; 2 Department of Computer Science, Princeton University, Princeton, USA; 3 Max Planck Institute for Empirical Aesthetics, Frankfurt am Main, Germany; 4 Department of Psychology, Cornell University, Ithaca, USA

**Keywords:** Human behaviour, Psychology

## Abstract

Determining the extent to which the perceptual world can be recovered from language is a longstanding problem in philosophy and cognitive science. We show that state-of-the-art large language models can unlock new insights into this problem by providing a lower bound on the amount of perceptual information that can be extracted from language. Specifically, we elicit pairwise similarity judgments from GPT models across six psychophysical datasets. We show that the judgments are significantly correlated with human data across all domains, recovering well-known representations like the color wheel and pitch spiral. Surprisingly, we find that a model (GPT-4) co-trained on vision and language does not necessarily lead to improvements specific to the visual modality, and provides highly correlated predictions with human data irrespective of whether direct visual input is provided or purely textual descriptors. To study the impact of specific languages, we also apply the models to a multilingual color-naming task. We find that GPT-4 replicates cross-linguistic variation in English and Russian illuminating the interaction of language and perception.

## Introduction

Imagine that you were chosen to be part of an expedition aimed at studying a newly discovered alien species on a distant planet. Your task is to understand the perceptual system of that species. You arrive at the planet and, to your dismay, discover that its inhabitants are long departed and the only thing they left behind is a huge archive of text. How much of the perceptual world of that species can you recover based on text alone? More concretely, how well can you recover the perceptual representations of that species based on the associations found in their text? Versions of this question have occupied philosophers for centuries^1–4^, and decades of psychological research are beginning to provide glimpses into the rich perceptual content of language and its influence on perception^5–13^.

But how can the amount of information that language provides about perception be quantified? Put differently, how well can we quantitatively capture perceptual judgments based on the statistical regularities of language? Here we propose to do so by eliciting perceptual judgments from large language models (LLMs) such as GPT-3 and its “ChatGPT” variants GPT-3.5 and GPT-4^14,15^. These models are trained on massive text corpora reflecting a substantial chunk of human language and can be queried in a way that is analogous to humans. They are also explicitly trained to learn the statistical regularities found in text by predicting masked tokens in context. Moreover, GPT-4 is further equipped with multimodal capabilities allowing it to process direct sensory data such as images, allowing for a nuanced analysis across multiple modalities.

Evaluating the extent to which LLMs can perform various cognitive tasks has drawn considerable interest recently, including domains such as language processing in the brain^16–18^, analogical reasoning^19^, perception^20–22^, cross-modal alignment^23^, and morality^24,25^. While the extent to which these models succeed in capturing human behavior vary across domains, when they do succeed robustly they provide an opportunity to ask how they do so given the human data they are trained on and the processing they deploy^16,17,19^. Likewise, some failures can be diagnostic of the gap between current LLM-based artificial intelligence and human intelligence^26,27^. Here, we use the fact that LLMs are trained on large amounts of language to gain insight into the classic problem of the relationship between language and perception: these models provide a lower bound on the amount of information about perceptual experience that can be extracted from language. It is a lower bound in the sense that human linguistic experience cannot be simply reduced into written text which forms the bulk of the training data of LLMs, and that human learning is presumably more than just learning associations via next-word predictions. More broadly, humans have direct access to the sensory world and can actively interact with the environment and one another. This kind of experience is absent from LLMs. Thus, by querying these models on various sensory judgment tasks, we can see how well they succeed in quantitatively capturing human judgments based on the text data they are trained on, and how well those judgments align with the perceptual representations of humans.

**Fig. 1:**
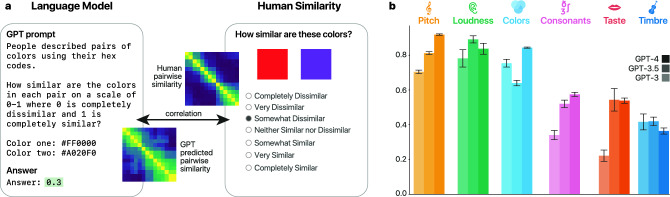
(**a**) Schematic of the LLM-based and human similarity judgment elicitation paradigms. (**b**) Correlations between models and human data across six perceptual modalities, namely, pitch, loudness, colors, consonants, taste, and timbre (Pearson *r*; 95% CIs).

We explored this empirically across six modalities, namely, pitch, loudness, colors, consonants, taste, and timbre. Given a stimulus space (e.g., colors) and its stimulus specification (e.g., wavelengths or some approximate hex-codes) we elicit pairwise similarity judgments in a direct analogy to the widespread paradigm of similarity in cognitive science^28^ using a carefully crafted prompt that is given to the model to complete (Fig. 1a; see “Methods”). Importantly, whereas four of the modalities were based on classical results from the literature (colors^29^, loudness^30^, timbre^31^ and taste^32^), two human datasets (pitch and vocal consonants) were novel and thus were not part of the training set of the models.

As noted above, unlike the other models GPT-4 was trained in a multimodal approach, enabling it to access both written text (similar to the other two variants) and images. This allowed us to examine if the additional sensory information resulted in enhanced performance in the color modality relative to the other domains, and to interrogate the underlying mechanisms that drive model predictions from language by analyzing performance on different sources of textual and visual input.

Finally, to further interrogate the impact of specific languages on the LLMs’ judgments, we tested whether they would behave differently in the presence of the same sensory information (a color hex-code), but respond in different languages. To that end, we conducted a color-naming task using a paradigm similar to that of Berlin and Kay^33^ and the World Color Survey^34,35^, and constructed human and GPT-3, GPT-3.5, and GPT-4 color-naming maps in both English and Russian.

## Results

### Similarity study

For each dataset, we designed a tailored prompt template that could be filled in with in-context examples and the pair of target stimuli for which we would want the LLM to produce a similarity rating (see “Methods” and SI for full specification of the prompts and datasets as well as additional controls for the choice of examples). Across all domains, we elicited 10 ratings per pair of stimuli from each of the GPT models and then constructed aggregate similarity ratings by averaging. We then evaluated the resulting scores by correlating them with human data. The Pearson correlation coefficients between human data and model predictions are shown in Fig. 1b (see “Methods” and SI for details regarding computing the correlations and CIs). We see that across all domains, the correlations were significant, and were particularly high for pitch ($r=.92$, 95% CI [.91, .92] for GPT-4), loudness ($r=.89$, 95% CI [.87, .91] for GPT-3.5), and colors ($r=.84$, 95% CI [.84, .85] for GPT-4) (and $>.6$ for all models), followed by moderate but highly significant correlations for consonants ($r=.57$, 95% CI [.56, .59] for GPT-4), taste ($r=.54$, 95% CI [.48, .61] for GPT-3.5) and timbre ($r=.42$, 95% CI [.40, .44] for GPT-3.5). For the two modalities for which we collected data, we could compare model performance to the inter-rater split-half reliability (IRR). The IRRs for pitch and consonants were $r=.95$ (95% CI [.94, .96]) and $r=.63$ (95% CI [.53, .73]), respectively, suggesting that the performance of GPT-4 is on par with human performance (see SI for additional experiments with an open-source LLM, Mistral-7B^36^, as well as embedding models).

We also note that in five out of the six domains, GPT-4 was among the top two models (this is also true when the open-source model is taken into account; see SI). Interestingly, the improvement relative to the average performance of the other models occurred across all modalities with the exception of timbre and loudness, and was not restricted or particularly large for the domain of colors (compare e.g. $\Delta r =.15$ 95% CI [.13, .16] for colors vs. $\Delta r =.16$ 95% CI [.15, .17] for pitch, $\Delta r =.16$ for taste 95% CI [.12, .19], and $\Delta r =.14$ 95% CI [.12, .16] for consonants) suggesting that this improvement is driven by richer textual training in GPT-4 rather than the possibility of its inclusion of images in its training set.

**Fig. 2:**
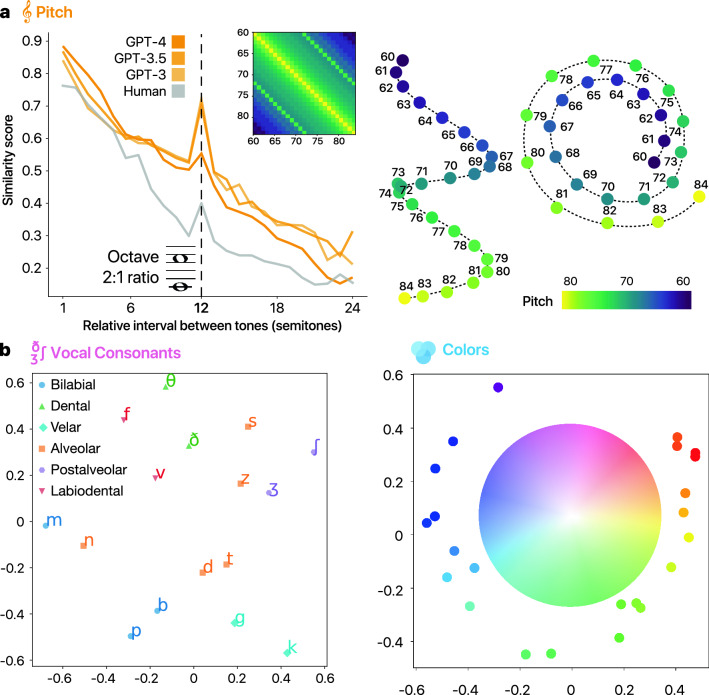
(**a**) Human and LLM similarity marginals and an example GPT-3 corresponding similarity matrix and its three-dimensional MDS solution for pitch. (**b**) MDS solutions for vocal consonants and colors for GPT-4 similarity matrices. To illustrate the structure of the results, we highlighted consonants with the same place of articulation in the vocal tract with the same shape and color, and added a rotated HSV color wheel for the color MDS.

Next, to get a finer picture of the LLM-based judgments and see to what extent they reflect human representations, we performed the following analyses. Starting from the domain of pitch, we wanted to see to what extent the LLM data captures a well-known psychological phenomenon that Western listeners tend to associate particular musical intervals or ratios of frequencies (such as the octave or 2:1 frequency ratio) with enhanced similarity^37^. To test this we computed the average similarity score over groups of pitch pairs that are separated by the same fixed interval (i.e., the same frequency ratios). Figure 2a shows the resulting average similarity per interval for the models and humans along with an example corresponding smoothed similarity matrix for GPT-3 (smoothing was done by averaging the raw similarity matrix over its sub-diagonals). We can see that apart from the decay as a function of separation (i.e., tones that are far apart in log frequency are perceived as increasingly dissimilar) there is a clear spike precisely at 12 semitones (octave), consistent with the aforementioned phenomenon of “octave equivalence”^38^. Moreover, applying multi-dimensional scaling^28^ (MDS) to the smoothed similarity matrix whereby the different stimuli are mapped into points in a Euclidean space (also known as “psychological space”) such that similar stimuli are mapped to nearby points reveals a clear helical structure with twists that correspond to precisely 12 semitone separations (i.e., octaves) recovering the pitch spiral representation (Fig. 2a). Likewise, applying MDS to the domains of consonants and colors (Fig. 2b) reveals highly interpretable representations, namely, the familiar color wheel and a production-based representation for consonants (see SI for additional controls in GPT-4 showing that the predictive power as well as the color-wheel MDS solution persist when other common color notations are used, namely, CIELAB and and CIELUV; Supplementary Fig. S1).

**Fig. 3:**
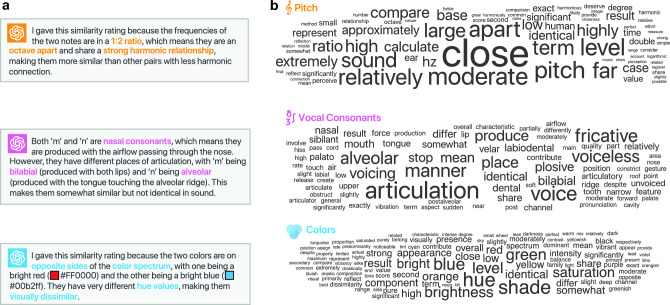
(**a**) Example GPT-4 explanations for similarity judgment scores. (**b**) Word clouds for GPT-4 explanations in the domain of pitch, vocal consonants, and colors.

As an additional analysis, we asked GPT-4 to provide explanations for the judgments it made (Fig. 3). We found that the model resorted to explanations involving the octave, ratios, and harmonic relations for pitch, places of articulation in the vocal tract for consonants, and hue, brightness and color spectra for colors, consistent with the MDS solutions and suggesting that the model’s behavior coheres at the quantitative and qualitative levels. More concretely, in the case of colors, some of the most common terms (excluding terms that were part of the prompt or are generic such as “similar” or “identical”) were *blue* (appeared in 49% of explanations), *green* (42%), *brightness* (36%), *hue* (35%), *saturation* (32%), *shade* (27%), and *red* (21%). As for pitch, these were *close* (41%), *higher* (15%), *ratio* (14%), *lower* (10%), *ear* (5%), *time* (5%), and *exact* (4%). Likewise, for consonants these were *articulation* (78%), *manner* (61%), *fricative* (51%), *voiced* (49%), *voiceless* (46%), *alveolar* (38%), and *bilabial* (27%). We also found that in the case of pitch, the model was attuned to special octave relations. Indeed, in 10 out of the 13 comparisons involving a 2:1 ratio between the first and second frequencies the model correctly resorted to explanations based on the octave construct.

Next, to better understand the linguistic mechanisms underlying model predictions we performed a series of control experiments. First, observe that the domain of colors is unique in that it can be analyzed in a fully multimodal fashion using the publicly available API of GPT-4(V) which allows for prompt-based processing of both images and text. This way we can see how predictions based on different forms of textual descriptors compare against direct visual judgments on color images. Specifically, we wanted to see to what extent the predictive power is driven by (i) direct sensory information, (ii) simple mathematical manipulations derived from the hex-code representation, and (iii) higher-order word associations^13^. To that end, we computed color similarity with GPT-4 based on the following complementary sets of input information that substitute the original hex-code values (see “Methods” and SI), (i) color pair images: images of color pairs were produced for each corresponding pair of color hex-codes, (ii) individual color terms: for each color hex-code we searched for a standard HTML color term that is as close as possible without imposing a one-to-one mapping between terms and codes (see “Methods”) to prevent reliance on specific hex-code manipulations (i.e., the model can no longer compute Euclidean distance between specific hex code values by converting them into perceptual color space coordinates), and (iii) sets of word associations that humans experience when a given color is observed: if the model indeed relies on such second-order information then we hypothesized that substituting the original stimuli with their indirect associations should still yield significant correlations (though possibly lower than the more accurate individual descriptors which is expected). Now, since such associations are difficult to extract from the training set of GPT-4, we elicited them directly from humans using a semantic mining framework known as STEP-tag^23^ whereby participants collaboratively describe stimuli using simple tags and evaluate tags provided by others (see “Methods” and SI). In previous research^23^, we demonstrated that the method is efficient and reliable at extracting high-quality verbal labels without relying on a fixed list of a priori associations. We found that all three input forms yielded significant correlations with human data, namely, $r=.77$ for direct images (due to rate limits on the number of multimodal API calls, we could not bootstrap that correlation stochastically and instead used a zero temperature prediction), $r=.79$, 95% CI [.78, .80] for color terms, and $r=.53$, 95% CI [.52, .54], for word associations. As a baseline, recall that the correlation with human data based on hex-codes was $r=.84$, 95% CI [.84, .85]. These results support the following conclusions: (i) eliciting similarity based on color-names or hex-codes performs on par with direct visual similarity which suggests that textual information is sufficient to drive accurate predictions, (ii) simple mathematical manipulations on hex-code representations are not necessary for producing high human correlations (though they may slightly boost the model performance as the hex-code-based predictions achieved the highest score), and (iii) replacing colors with high-level associations still yield significant predictive power which implies that they indirectly contribute to color similarity.

The second control concerned the pitch domain. Note that while our pitch data is new and as such could not have been part of the training set of any of the models, the fact that we considered frequency values that correspond to common discrete MIDI notes (60–84, or C4–C6) could still allow for some reliance on memory, e.g., by accessing frequency-to-MIDI conversion tables. To control for that, we jittered the frequency values by randomly adding ± 0.1 semitones. This small noise may weaken the strength of the octave peaks to some extent (as expected from de-tuning effects on pitch and melody representations^39^) but it should not have any drastic effects on the general structure. If, on the other hand, the model relies largely on lookup tables of MIDI note conversion then the predictive power should be significantly compromised as the jittered values no longer correspond to any definite MIDI note values. We found that the former was the case, namely, that the correlations indeed remained quite high ($r=.86$, 95% CI [.85, .87], for GPT-3; $r=.89$, 95% CI [.88, .90], for GPT-3.5; $r=.95$, 95% CI [.94, .95], for GPT-4). These values are in fact slightly higher than before, possibly because the original exact values tended to produce somewhat exaggerated profiles relative to the human baseline (Fig. 2a and Supplementary Fig. S2).

### Color naming study

The results so far suggest that LLMs can use textual information to form perceptual representations. If this is indeed the case, we hypothesized that different languages of the prompt may influence the judgments of the LLMs in the presence of identical input. This would be consistent with cross-cultural differences observed in humans suggesting that different languages lead to different partitioning of a given perceptual space^6,34,40^, as well as previous claims in the literature concerning the influence of language on perceptual representations^8,41–43^. To test this, we propose to test LLMs on an explicit naming task introduced in the seminal work of Berlin and Kay^33^ and further explored across cultures around the globe^34,35^, and using computational modeling^6,9^. It is also worth noting that color naming was studied recently in the context of previous-generation language models^44^, as well as other artificial agents^45,46^.

**Fig. 4:**
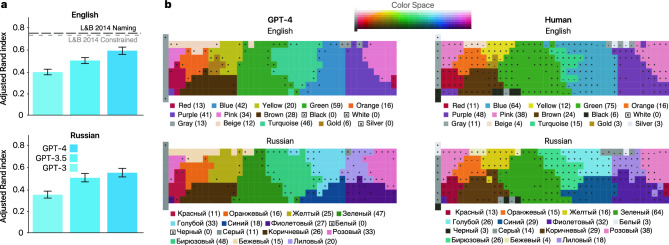
Color naming experiment using 330 Munsell colors from the World Color Survey (top, color space). (**a**) Adjusted Rand index illustrating the alignment between human and LLM experiments (95% CIs). The dashed lines for English represent lab-based free naming and forced-choice naming experiments collected by Lindsey and Brown^35^ (data reproduced with permission). (**b**) Data comparison between humans and LLMs in Russian and English. Participants and LLMs were shown colors and were asked to choose from the same 15-color list. The count of chosen colors for each option is given in parentheses. The color of a response cluster in the maps represents its average color (see Supplementary Fig. S3 for all maps). Colors for which less than 50% and 90% of the times the dominant color term was selected were indicated by “−” and “*”, respectively. If the dominant color term was selected more than 90% of the time, no marking was used.

We thus tested whether LLMs would yield different naming patterns of color hex codes depending on the language of the prompt used to elicit those names (see “Methods”; Fig. 4 and Supplementary Fig. S3). Specifically, we presented both humans and LLMs with different colors and asked them to perform a forced-choice naming task by selecting from a pre-specified list of 15 color names (see “Methods”). We specifically focused on English and Russian as test cases, since Russian speakers are documented to use richer vocabulary to describe what English speakers would otherwise describe as blue and purple^33,43,47^. We collected data from 103 native English speakers and 51 native Russian speakers and compared them against LLMs performing the same task, and to the in-lab data of Lindsey and Brown^35^ as an additional baseline.

The results are shown in Fig. 4 (additional results for an open-source model are provided in SI). Our first finding was that GPT-4 maps in both English and Russian were more human-like than the other variants when compared with an adjusted Rand index (see Fig. 4a; English: GPT-4 0.59 95% CI [0.56, 0.63], GPT-3.5, 0.50 95% CI [0.46, 0.52], GPT-3 0.39 95% CI [0.37, 0.42]; Russian: GPT-4 0.54 95% CI [0.46, 0.54], GPT-3.5 0.50 95% CI [0.45, 0.52], GPT-3 0.35 95% CI [0.29, 0.35]; see “Methods”). It is evident from our data, however, that LLMs are still not perfect in predicting human color naming as compared to a separate lab-based experiment conducted by Lindsey and Brown^35^ (dashed line in Fig. 4a top, constrained naming task 0.73 95% CI [0.65,0.74], free naming task 0.75 95% CI [0.66,0.75]). Likewise, unlike the similarity domain, we found in an additional control experiment (see SI) that GPT-4’s performance in the naming task is compromised when using CIELAB/CIELUV color coordinates instead of hex (see Discussion). Moreover, the naming of GPT-4 colors differs from human data in some important cases, including the color turquoise, which was selected as the dominant color for 46 Munsell colors in GPT-4 versus only 15 in human data. Note, however, that our human English results conducted online and with relative less control over color presentation were highly consistent with those of Lindsey and Brown^35^ that were conducted in the lab and under controlled environment, even though Lindsey and Brown^35^ used slightly different paradigms: free naming (consistency to our human data: 0.75 95% CI [0.66, 0.75]) and forced-choice list with a different set of items (0.73 95% CI [0.65, 0.74]).

Importantly, however, GPT-4 appears to replicate cross-lingual differences (Fig. 4b), for example separating Russian blue and purple into distinct categories for lighter and darker areas^43^. Indeed, the color sínij / синий (Blue) was the dominant category for 18 and 29 Munsell colors for GPT-4 and humans, respectively, and the color golubój / голубой (Light-blue) was accordingly the dominant category for 33 and 26 colors. Similarly, the color fiolétovyj / фиолетовый (Violet) was the dominant category for 27 and 32 Munsell colors for GPT-4 and humans, respectively, and the color lilóvyj / лиловый (Lilac) was accordingly the dominant category for 20 and 18 Munsell colors (see Supplementary Figure S3 for similar light and dark blue distinctions in GPT-3.5 which further suggest that such distinctions can be learned without visual input training).

## Discussion

In this work, we showed how recent advances in large language models and, in particular, their flexible prompt engineering capabilities provide an elegant way for extracting clear quantitative psychophysical signals from text corpora. Our results contribute to a variety of thought-provoking issues in perception and language research. In particular, our findings further support recent research suggesting that people that lack direct sensory experiences (e.g., congenitally blind individuals) could still possess a rich understanding of perceptual concepts through language (e.g., colors^13^ or appearance^10,12^). Our control analyses also shed light on the underlying mechanisms that drive this perceptual knowledge and situate them, in part, in direct and higher-order semantic associations consistent with distributional statistics accounts of visual knowledge^12,13^. Likewise, the language-dependence of the color-space partitions of GPT-4 in the naming task suggests that the physical stimulus alone (in our case, the color hex code) is insufficient for explaining the behavioral patterns of the model since the same hex codes triggered different color categorizations in Russian and English. This finding is consistent with two possible interpretations. One possibility is that language truly distorts the perceptual representation. This is consistent with prior work suggesting that performance in low-level discrimination tasks is impacted by the speaker’s language (e.g., native Russian speakers were shown to be faster at discriminating different shades of blue when the colors fell into different linguistic categories in Russian as opposed to colors from the same category; native English speakers tested on the same stimuli did not exhibit this category advantage^43^). Alternatively, our results could reflect different color-name mappings in different languages with respect to the same underlying perceptual representation. This is consistent with previous literature that demonstrates that cross-cultural variation in color naming maps can be accounted for by optimal partitions of the same perceptual space with different numbers of terms^6,9^. Finally, the fact that GPT-4 achieved IRR-level performance without any fine-tuning in the newly collected datasets of pitch and vocal consonants, contributes to our understanding of LLMs’ ability to mimic human behavior^15^.

We end by discussing some limitations which point towards future research directions. First, while in our work we experimented with three different types of behavioral data (similarity, explanations, and naming), there are other perceptual measures that one could consider (for example, odd-one-out triplet judgments^48^). Future work could explore these measures and interrogate to what extent they too yield a consistent representation. Second, our work is restricted to population-level averages as a leading-order analysis, a follow-up study could look into the natural variability of the LLM judgments and see to what extent they capture individual-level differences in humans. Third, our treatment of color and pitch perception using single wavelength or frequency measures is naturally an idealization. Indeed, colored light produced from computer monitors is not monochromatic, and musical tones are often composed of an array of harmonic partials. Nevertheless, the fact that our text-based similarity judgments yielded high correlations with human data, and the fact that we observed high agreement with in-lab studies in the case of color naming experiments suggest that this approximation is a useful one when it comes to modeling perceptual judgments using LLMs. Fourth, while our work provides some evidence for LLMs’ ability to capture cross-cultural differences, it remains to be seen how far this holds for other languages, especially those that are underrepresented^41^. Relatedly, while we found that GPT-4’s similarity predictions were robust to the choice of color coordinates, this was not the case in the color naming experiment where the performance was compromised (see SI). While a modification of the prompt could potentially remedy this (e.g., by explicitly instructing the model to convert to hex-codes), this observation suggests that the source of training data that the model relies on to perform the naming task is much more heavily based on hex-codes as these are very common in HTML documents that are part of the model’s training set. Future work could investigate how the inclusion of more balanced training data could improve this behavior. Finally, it is important to point out that the very same massive training pipelines that make LLMs a powerful proxy of human language also make them particularly susceptible to inheriting biases^49^. Researchers should be particularly cautious when interpreting the patterns of behavior elicited from LLMs and should always benchmark them against genuine human data. To conclude, our work showcases how LLMs can be used to explore the limits of what information about the world can be recovered from language, and more broadly, it highlights the potential of combining human and machine experiments in order to address fundamental questions in cognitive science.

## Methods

### GPT prompt elicitation

The general structure of the prompt elicitation template for the similarity experiments was: one sentence describing the dataset (e.g., *“people described pairs of colors using their hex code.”*), one sentence describing the similarity rating task and scale (e.g., *“how similar are the colors in each pair on a scale of 0-1 where 0 is completely dissimilar and 1 is completely similar?”*), three sets of three lines each corresponding to two stimuli and their actual similarity rating taken from behavioral experiments which serve as in-context examples, and an additional set of three lines corresponding to the pair of target stimuli and an associated empty rating field for the model to fill in (*“Color one: #FF0000. Color two: #A020F0. Rating:”*). These were necessary to ensure that the model provided numerical values, and in all cases consisted of three fixed and randomly chosen comparisons so that most of the content was left for the model to produce. For each pair of target stimuli, we elicited ten ratings from each GPT model. Across all repetitions of all pairs of stimuli for a given dataset, we used only the same three in-context examples to ensure that the model is exposed to only a very small fraction of the similarity judgments against which its ratings were compared (see SI for full prompts and additional details).

For the multimodal color similarity experiments we used the vision variant of GPT-4 (GPT-4V) by generating an image for each pair of colors that consisted only of two squares, side-by-side, corresponding to the two colors being compared in the pair. For each pair, we provided the corresponding image along with the same single sentence prompt as above. We did not add the additional examples provided in other experiments as this would require multiple images to be sent with each prompt. As a result, GPT-4V results are zero-shot results (i.e., it is likely that they would be even better in the in-context example case). Due to very low GPT-4V API rate limits we could elicit only a single rating per pair, which we elicited at zero temperature to recover the highest probability response in each case.

For the color-naming experiments, we first elicited 15 basic color names from GPT-4 using the prompt *“Name 15 basic colors.”* and a temperature of 0 (to get the highest probability answers). We then had GPT-4 name the hex code corresponding to each of the WCS colors using the following prompt: *“Here is a list of 15 basic color names: <shuffled basic color list>. Which of these names best describes the following color: <hex-code>? Respond only using the name.”* We repeated this prompt ten times for each WCS color with the basic color list shuffled each time and temperature set to the default 0.7 to elicit ten names per WCS color. We repeated the full procedure with both prompts translated to Russian (GPT-4 also responded to both of these in Russian; see SI for full prompt and additional details).

### Stimuli

The six human similarity datasets we considered come in two flavors—direct (dis-)similarity ratings and confusion matrices—and from two sources—previous psychological studies from the literature and newly collected datasets. Confusion matrices provide an alternative way to compute similarity scores between stimuli by counting the number of times a stimulus *x* is confused for a stimulus *y*. By normalizing the counts one gets confusion probabilities $p_{xy}$ which can be converted into similarity scores using the formula $s_{xy}=\sqrt{p_{xy}p_{yx}/p_{xx} p_{yy}}$^50,51^.

#### Colors

This dataset was taken from^29^ (also reproduced in^28^) and comprised direct similarity judgments across a set of 14 colors with wavelengths in the range $434-674$ nanometers. We converted wavelengths into RGB using the script at https://hasanyavuz.ozderya.net/?p=211 (also provided in the code repository; see Code availability below) and then we used the webcolors Python package to convert into hex codes. To get better coverage of the color wheel in Fig. 2b we extended the space to 23 color stimuli by interpolating between the original colors in the dataset and eliciting an extended similarity matrix from GPT-4.

#### Pitch

This dataset was collected and made publicly available very recently by a subset of the authors in^52^ (see details below). It contains similarity judgments over pairs of 25 harmonic complex tones (10 partials and 3dB/octave roll-off) over a two octave range from C4 (60 MIDI; 261.626 Hz) to C6 (84 MIDI; 1046.502 Hz). The pitch values were separated by 1 semitone steps to account for the fact that pitch perception is logarithmic^38^ where the mapping between frequencies *f* in Hertz and pitch *p* in semitones are given by $p=12 \log _2{f/440} + 69$.

#### Vocal consonants

This dataset was also collected via an online study (see below) and comprised similarity judgments over 16 recordings of vocal consonants taken from the International Phonetic Association. The vocal consonants considered were b (**b**ay), p (**p**ay), m (**m**ay), n (**n**o), ɡ (**g**o), k (ca**k**e), d (**d**ie), t (**t**ie), f (**f**ee), v (**v**ow), s (**s**o), Ɵ (**th**igh), ð (**th**ey), Ʒ (**J**acques), and ʃ (**sh**ow). The recordings came from two speakers, one male and one female.

#### Loudness

We accessed this dataset via^51^ which itself takes the data from^30^. The dataset comes in the form of a confusion matrix over 8 pure tones of different loudness values ranging from 71.1 to 74.6 decibels.

#### Taste

This dataset was also accessed via^51^ and is taken from^32^. The data comes in the form of a confusion matrix over 10 flavors described to participants as salt, salt-substitute, MSG, quinine, acid, sugar, artificial sweetener, salt-sugar, acid-sugar, and quinine-sugar.

#### Timbre

This dataset was assembled in^31^ based on 1217 subject’s judgments from 5 prior publications. It comprises dissimilarity judgments over 12 instrument timbres: clarinet, saxophone, trumpet, cello, French horn, oboe, flute, English horn, bassoon, trombone, violin, and piano.

### Behavioral experiments

To collect similarity judgments over pitch and vocal consonants, we deployed two online experiments on Amazon Mechanical Turk (AMT). Overall, 55 participants completed the pitch study, and 64 participants completed the vocal consonants study. To collect color word associations, we recruited 17 UK participants from Prolific (https://www.prolific.com). Finally, for the color naming study, we collected data from Russian and British English participants through Prolific: overall, we recruited 154 participants, of whom 103 were UK participants and 51 were Russian participants. Experiments were implemented using PsyNet (https://psynet.dev/) and Dallinger (https://dallinger.readthedocs.io/). All participants provided informed consent in accordance with approved protocols by the Princeton Institutional Review Board (#10859) and the Max Planck Ethics Council (#2021_42). See additional details below.

#### Similarity experiments: participants

As noted above, 55 participants completed the pitch study and 64 participants completed the vocal consonants study. Participants were recruited from the United States, were paid $9-12 USD per hour, and provided informed consent as approved by the Princeton IRB (#10859) and the Max Planck Ethics Council (#2021_42). The recruitment and experimental pipelines were automated using PsyNet^53^, a modern framework for experiment design and deployment which builds on the Dallinger platform for recruitment automation.

To enhance data quality, participants had to pass a standardized headphone check^54^ that ensures good listening conditions and task comprehension, and were required to have successfully completed at least 3000 tasks on AMT. Upon passing the prescreening stage, participants were randomly assigned to rate the similarity between different pairs of stimuli and provided numerical judgments on a 7-Likert scale ranging from 0 (completely dissimilar) to 6 (completely similar). In the pitch experiment, participants provided an average of 80 judgments, and in the vocal consonants experiment an average of 55 judgments. Inter-rater reliability was estimated using a split-half method bootstrapped over participants with a Spearman-Brown correction^55^ $2r/(1+r)$.

#### Similarity experiments: procedure

Upon providing informed consent and passing the headphone check, participants received the following instructions. In the case of the pitch experiment: “In this experiment we are studying how people perceive sounds. In each round you will be presented with two sounds and your task will be to simply judge how similar those sounds are. You will have seven response options, ranging from 0 (‘Completely Dissimilar’) to 6 (‘Completely Similar’). Choose the one you think is most appropriate. You will also have access to a replay button that will allow you to replay the sounds if needed. Note: no prior expertise is required to complete this task, just choose what you intuitively think is the right answer.” Participants were then informed of an additional small quality bonus “The quality of your responses will be automatically monitored, and you will receive a bonus at the end of the experiment in proportion to your quality score. The best way to achieve a high score is to concentrate and give each round your best attempt”. While the task is subjective in nature, we used consistency as a proxy for quality by repeating 5 random trials at the end of the experiment and computing the Spearman correlation *s* between the original responses and their repetitions. The final bonus was computed using the formula $\min (\max (0.0, 0.1s),0.1)$ yielding at most 10 cents. In the main experiment participants were assigned to random stimulus pairs and were instructed to rate their similarity using the following prompt: “How similar are the pair of sounds you just heard?” and provided a response on a Likert scale. The procedure for the vocal consonants similarity experiment was identical up to the specific instructions. Specifically, participants received the following instructions: “In this experiment we are studying how people perceive the sound of vocal consonants. A consonant is a speech sound that is pronounced by partly blocking air from the vocal tract. For example, the sound of the letter *c* in *cat* is a consonant, and so is *t* but not *a*. Similarly, the sound of the combination *sh* in *sheep* is a consonant, and so is *p* but not *ee*. In general, vowel sounds like those of the letters *a, e, i, o, u* are not consonants.” The instructions then proceeded: “In each round you will be presented with two different recordings each including one consonant sound and your task will be to simply judge how similar are the sounds of the two spoken consonants. We are not interested in the vowel sounds nor in the voice height, just the sound of the consonants. You will have seven response options, ranging from 0 (‘Completely Dissimilar’) to 6 (‘Completely Similar’). Choose the one you think is most appropriate. Note: no prior expertise is required to complete this task, just choose what you intuitively think is the right answer.” Participants were then informed of the quality bonus which was identical to the pitch task, and then rated the similarity between pairs of random consonants based on the following prompt “How similar is the sound of the consonants pronounced by the two speakers?” and a Likert scale as before.

#### Similarity experiments: model evaluation

We quantified model performance in predicting human similarity judgments by computing the Pearson correlation coefficient between the flattened upper triangle of the LLM-based and human-based similarity matrices (to account for the fact that these matrices are symmetric). This approach is similar to representational similarity analysis^56^. To compute 95% confidence intervals, we bootstrapped with replacement over model predictions with 1,000 repetitions and computed for each repetition the average similarity matrix. We then correlated the upper triangles of each of those matrices with human data to produce a list of correlation coefficients on which we computed confidence intervals.

#### STEP-Tag control: experiment

To elicit human impressions of colors, we used a recently developed adaptive tag mining pipeline called Sequential Transmission Evaluation Pipeline^23^ (STEP-Tag). In this paradigm, participants adaptively annotate a set of target stimuli (here, color patches), both by providing new descriptive tags for the stimulus and by simultaneously rating the tags produced by previous participants. Participants also have the possibility of flagging tags they deem inappropriate. Tags are removed if they are flagged twice (but can potentially reappear if a future participant adds them again). As the process unfolds over multiple iterations, meaningful tags emerge that describe the stimulus well and are validated by multiple participants, thus enabling free elicitation of tags describing the stimulus. This paradigm has been shown to be effective in eliciting open-ended taxonomies without pre-specification across multiple modalities, and in capturing perceptual and semantic similarity in the representation of humans and deep learning models^23^.

In the present experiment, 17 participants annotated 14 color patches. Each color, on average, received ten iterations. Each participants observed ten color patches. For each tagging process, we extracted the tags at the final iteration. These tags were used for further analysis. The elicited tags can be accessed through the Data availability section.

#### STEP-Tag control: analysis

After extracting the tags from the final iteration of the procedure described above, we filtered out any “low-rated” tags (those with an average rating less than 3). No additional pre-processing was applied to any individual tags (i.e., some tags may have different spellings or typos). Tags for each individual color were joined into a comma-separated list, and these lists were then used to elicit similarity rating from GPT model using a slightly modified version of the previously used color similarity judgment prompts (see SI for full prompt).

#### Color term control: LLM similarity elicitation

We converted color hex-codes to HTML color terms using the following online tool https://www.color-name.com/hex and checked that there is indeed no one-to-one mapping between terms and codes (by exploring different hex code values). The chosen list of color names was: electric ultramarine, blue (RYB), azure, blue bolt, aqua, guppie green, lawn green, bitter lime, yellow rose, amber, vivid gamboge, orange (pantone), red, red (see SI for full prompt).

#### Color naming experiments: participants

To collect the color naming data in Russian and British English participants, we ran online experiments on Prolific. Overall, we recruited 103 UK participants and 51 Russian participants. All texts in the interface of the experiment (e.g., buttons, instructions, etc.) were presented in the native language of the participant. The Russian texts were first automatically translated using DeepL (https://www.deepl.com) and then manually checked and corrected by a native speaker of Russian (author I.S). Participants had to be raised monolingually and to speak the target language as their mother tongue. Each participant was paid 9 GBP per hour and provided informed consent according to an approved protocol (Max Planck Ethics Council #2021_42). The experiment was implemented using PsyNet^53^.

#### Color naming experiments: procedure

The color naming experiments consisted of two stages. In the first stage we freely elicited basic color terms. Participants received the following instructions: English: “Please name at least 8 basic color names. Press enter after each color name. Only use lower-case letters.”, Russian: “Укажите не менее 8 названий основных цветов. Нажмите клавишу Enter после каждого названия цвета. Используйте только строчные буквы”. Participants could only submit color names without spaces, numbers, or special characters and may only submit the page if they have provided at least eight names. The list of obtained colors is highly overlapping with the GPT-4 list, justifying our choice to use GPT-4 as the basis for the word naming task (colors are sorted by their naming frequency). Specifically, the top 15 terms in English were:

“blue”, “green”, “yellow”, “red”, “purple”, “orange”, “black”, “pink”, “white”, “brown”, “grey”, “violet”, “indigo”, “turquoise”, “silver”

Likewise, the top 15 terms in Russian were:

“красный”, “синий”, “белый”, “зеленый”, “оранжевый”, “желтый”, “фиолетовый”, “черный”, “голубой”, “коричневый”, “розовый”, “серый”, “жёлтый”, “зелёный”, “чёрный”

From the top 15 color terms, 11 (English) and 12 (Russian) color terms are overlapping with the list provided by GPT-4 (for Russian the difference was simply due to accent variants in the list above). Next, in the second stage participants performed the color naming task. Before the main experiments, participants received the following instructions: English: “During this experiment, you will be presented with a square of a particular color and will be required to select the most suitable color term from a list of options. Please be aware that some of the colors may be repeated to verify consistency of your choices. If we detect any inconsistencies in your answers, we may terminate the experiment prematurely. The best strategy is to answer each question truthfully, as attempting to memorize responses may prove difficult.”, Russian: “В ходе этого эксперимента вам будет представлен квадрат определенного цвета, и вам нужно будет выбрать наиболее подходящее название цвета из списка вариантов. Имейте в виду, что некоторые цвета могут повторяться, чтобы убедиться в согласованности вашего выбора. Если мы обнаружим какие-либо несоответствия в ваших ответах, мы можем досрочно прекратить эксперимент. Лучшая стратегия - отвечать на каждый вопрос правдиво, так как попытка запомнить ответы может оказаться сложной”. The participants then went through the main experiment with the following prompts.

#### Color naming task prompts

English:

<square of a particular color>

You will see below a list of 15 basic color names. Which of these names best describes the color above?

<shuffled basic color list presented as buttons>

Russian:

<square of a particular color>

Ниже Вы увидите список из 15 основных названий цветов.

Какое из этих названий лучше всего описывает вышеуказанный цвет?

<shuffled basic color list presented as buttons>

At the end of the experiment, the participant took a color blindness test^57^. Some participants abandoned the experiment prematurely, but we nevertheless included their responses (42 English participants, 3 Russian participants). Only a fraction of the participants failed the color blindness test (5 of 103 English participants, and 2 of 51 Russian participants). Consistent with the WCS we included all participants including those who failed the color blindness test. In a control analysis, we excluded all color blind individuals and all participants that did not complete the entire session and got nearly identical results (the adjusted Rand index was 0.92 for English experiments and 0.97 for Russian experiments).

#### Color naming experiments: analysis

For each color, we collected at least 10 responses per LLM variant, and at least 10 forced-choice human selections per color (English mean 19.30 responses, Russian mean 12.17 responses). Consistent with previous literature for each Munsell’s color we selected the most frequently reported term. We then presented the dominant colors in Fig. 4b. To aid visualization we averaged the RGB values of all colors with the same color term, and presented them as the legend and clustered color in that figure. We also listed per color the number of Munsell colors that were associated with each dominant color term. Figure 4b provides additional information on the degree of agreement for each color. Colors for which less than 50% and 90% of the times the dominant color term was selected were indicated by “-” and “*”, respectively. If the dominant color term was selected more than 90% of the time, no marking was used.

#### Adjusted Rand index

The Rand index^58^ is a label-insensitive measure of clustering similarity that instead of relying on specific labels (e.g. “Blue”) quantifies the similarity between two clustering partitions by counting pairs of items (in our case Munsell colors) that are clustered consistently and dividing them by the overall number of pairs. This allows to compare different clustering schemes when the vocabulary of labels is not aligned (e.g. English and Russian). Formally, we computed: $R=(b+c)/a$; where *b* is the number of pairs of items that are in the same subset in one clustering and in the same subset in the other, *c* is the number of pairs of items that are in different subsets in one clustering and in different subsets in the other and *a* is the total number of pairs. The Rand index provides high values for two random clusterings, to adjust for this we used the corrected-for-chance version of the Rand index^58^, which normalizes the raw value by the expected value of the Rand index for random clusterings. Formally, we have $ARI = (RI - RI_{\text {rand}}) / (1 - RI_{\text {rand}})$ where *ARI* is the adjusted Rand index, *RI* is the raw Rand index and $RI_{\text {rand}}$ is the expected Rand index for random clusterings. The adjusted Rand index is thus ensured to have a value close to 0.0 for random labeling independently of the number of clusters and samples, exactly 1.0 when the clusterings are identical (up to a permutation), and reaches -0.5 for “orthogonal clusters” that are less consistent relative to what is expected by chance. In our case, all values were strictly positive suggesting consistency across languages and experiments. To compute confidence intervals, we created bootstrapped datasets by sampling the responses of each color with replacement and recomputing the dominant selected color name. We then obtained CIs by computing the adjusted Rand index for 1,000 pairs of bootstrapped datasets.

#### Lindsey and Brown dataset

We compared our experimental data to a dataset by Lindsey and Brown^35^, reproduced with permission by Delwin Lindsey. The data contains two experimental conditions conducted in the lab with the same 51 participants. In the first condition, participants were instructed to provide free naming responses. In the second condition, participants were instructed to choose from a pre-specified list of 11 color terms: (Green, Blue, Purple, Pink, White, Brown, Orange, Yellow, Red, Black, and Gray). Despite the fact that the Lindsey and Brown experiment was conducted in the lab (and not online like our experiments) and that the constrained list in our experiment was somewhat different, the results of both experiments were highly consistent with our human English data (Constrained, $ARI=0.73$ 95% CI [0.65, 0.74], free naming 0.75 95% CI [0.66, 0.75]). In addition to putting an upper bound on the consistency with which the LLM can predict human data (by comparing it with another human experiment), these results prove that despite less control over color presentation compared to the lab, online presentation still provides high-quality color naming data.

## Supplementary Information

Supplementary Information.

## Data Availability

All data used in this work can be accessed via the following link: https://github.com/computational-audition/LLM-psychophysics/tree/main, with the exception of the color naming dataset of Lindsey & Brown^35^ as it is only available upon request from the authors. An interactive visualization of raw similarity matrices and their two-dimensional MDS spaces for the six modalities is available at: https://computational-audition.github.io/LLM-psychophysics/all-modalities.html. The human color naming data can be interactively explored via: https://computational-audition.github.io/LLM-psychophysics/color.html.

## Code availability

All code used in the present work is available at the following repository: https://github.com/computational-audition/LLM-psychophysics/tree/main.

## References

[1] Forster, M. & von Herder, J.G. In *The Stanford Encyclopedia of Philosophy* (Zalta, E. N. & Nodelman, U. eds.). Winter 2023 Ed. (Metaphysics Research Lab, Stanford University, 2023).

[2] Hume, D. *An Abstract of a Treatise of Human Nature*. Vol. 1740 (CUP Archive, 1740).

[3] Locke, J. *An Essay Concerning Human Understanding* (Kay & Troutman, 1847).

[4] Koerner, E. F. Towards a ‘full pedigree’ of the ‘Sapir-Whorf hypothesis’: From Locke to Lucy. In *Explorations in Linguistic Relativity*. 1–24 (2000).

[5] Goldstone, R. L. & Rogosky, B. J. Using relations within conceptual systems to translate across conceptual systems. *Cognition* **84**, 295–320 (2002). 12044737 10.1016/S0010-0277(02)00053-7

[6] Regier, T., Kay, P. & Khetarpal, N. Color naming reflects optimal partitions of color space. *Proc. Natl. Acad. Sci.* **104**, 1436–1441 (2007). 17229840 10.1073/pnas.0610341104 PMC1783097

[7] Regier, T. & Kay, P. Language, thought, and color: Whorf was half right. *Trends Cognit. Sci.* **13**, 439–446 (2009). 19716754 10.1016/j.tics.2009.07.001

[8] Dolscheid, S., Shayan, S., Majid, A. & Casasanto, D. The thickness of musical pitch: Psychophysical evidence for linguistic relativity. *Psychol. Sci.* **24**, 613–621 (2013). 23538914 10.1177/0956797612457374

[9] Zaslavsky, N., Kemp, C., Regier, T. & Tishby, N. Efficient compression in color naming and its evolution. *Proc. Natl. Acad. Sci.* **115**, 7937–7942 (2018). 30021851 10.1073/pnas.1800521115 PMC6077716

[10] Kim, J. S., Elli, G. V. & Bedny, M. Knowledge of animal appearance among sighted and blind adults. *Proc. Natl. Acad. Sci.* **116**, 11213–11222 (2019). 31113884 10.1073/pnas.1900952116 PMC6561279

[11] Kim, J. S., Aheimer, B., Montané Manrara, V. & Bedny, M. Shared understanding of color among sighted and blind adults. *Proc. Natl. Acad. Sci.* **118**, e2020192118 (2021). 34385310 10.1073/pnas.2020192118 PMC8379969

[12] Lewis, M., Zettersten, M. & Lupyan, G. Distributional semantics as a source of visual knowledge. *Proc. Natl. Acad. Sci.* **116**, 19237–19238 (2019). 31488726 10.1073/pnas.1910148116 PMC6765286

[13] van Paridon, J., Liu, Q. & Lupyan, G. How do blind people know that blue is cold? Distributional semantics encode color-adjective associations. In *Proceedings of the Annual Meeting of the Cognitive Science Society*. Vol. 43 (2021).

[14] Brown, T. *et al.* Language models are few-shot learners. *Adv. Neural Inf. Process. Syst.* **33**, 1877–1901 (2020).

[15] OpenAI. *GPT-4 Technical Report*. 303.08774 (2023).

[16] Goldstein, A. *et al.* Shared computational principles for language processing in humans and deep language models. *Nat. Neurosci.* **25**, 369–380 (2022). 35260860 10.1038/s41593-022-01026-4 PMC8904253

[17] Kumar, S. *et al.* Reconstructing the cascade of language processing in the brain using the internal computations of a transformer-based language model. *BioRxiv* 2022-06 (2022).

[18] Tikochinski, R., Goldstein, A., Yeshurun, Y., Hasson, U. & Reichart, R. Perspective changes in human listeners are aligned with the contextual transformation of the word embedding space. *Cerebral Cortex* bhad082 (2023). 10.1093/cercor/bhad082 36939309

[19] Webb, T., Holyoak, K. J. & Lu, H. Emergent analogical reasoning in large language models. *Nat. Hum. Behav.* **7**, 1526–1541 (2023). 37524930 10.1038/s41562-023-01659-w

[20] Patel, R. & Pavlick, E. Mapping language models to grounded conceptual spaces. In *International Conference on Learning Representations* (2021).

[21] Siedenburg, K. & Saitis, C. How does chatgpt rate sound semantics? arXiv preprint arXiv:2304.07830 (2023).

[22] Zhang, C., Van Durme, B., Li, Z. & Stengel-Eskin, E. Visual commonsense in pretrained unimodal and multimodal models. arXiv preprint arXiv:2205.01850 (2022).

[23] Marjieh, R. et al. Words are all you need? capturing human sensory similarity with textual descriptors. In *The Eleventh International Conference on Learning Representations* (2022).

[24] Dillion, D., Tandon, N., Gu, Y. & Gray, K. Can AI language models replace human participants? *Trends Cognit. Sci.* (2023). 10.1016/j.tics.2023.04.008 37173156

[25] Ganguli, D. et al. The capacity for moral self-correction in large language models. arXiv preprint arXiv:2302.07459 (2023).

[26] McCoy, R. T., Yao, S., Friedman, D., Hardy, M. & Griffiths, T. L. Embers of autoregression: Understanding large language models through the problem they are trained to solve. arXiv preprint arXiv:2309.13638 (2023). 10.1073/pnas.2322420121 PMC11474099 39365822

[27] Binz, M. & Schulz, E. Using cognitive psychology to understand GPT-3. *Proc. Natl. Acad. Sci.* **120**, e2218523120 (2023). 36730192 10.1073/pnas.2218523120 PMC9963545

[28] Shepard, R. N. Multidimensional scaling, tree-fitting, and clustering. *Science* **210**, 390–398 (1980). 17837406 10.1126/science.210.4468.390

[29] Ekman, G. Dimensions of color vision. *J. Psychol.* **38**, 467–474 (1954). 10.1080/00223980.1954.9712953

[30] Kornbrot, D. E. Theoretical and empirical comparison of Luce’s choice model and logistic Thurstone model of categorical judgment. *Percept. Psychophys.* **24**, 193–208 (1978). 704280 10.3758/BF03206089

[31] Esling, P., Bitton, A. et al. Generative timbre spaces: Regularizing variational auto-encoders with perceptual metrics. arXiv preprint arXiv:1805.08501 (2018).

[32] Hettinger, T. P., Gent, J. F., Marks, L. E. & Frank, M. E. Study of taste perception. *Percept. Psychophys.* **61**, 1510–1521 (1999). 10598466 10.3758/BF03213114

[33] Berlin, B. & Kay, P. *Basic Color Terms: Their Universality and Evolution* (University of California Press, 1991).

[34] Kay, P., Berlin, B., Maffi, L., Merrifield, W. R. & Cook, R. *The World Color Survey* (Citeseer, 2009).

[35] Lindsey, D. T. & Brown, A. M. The color lexicon of American English. *J. Vis.* **14**, 17–17 (2014). 24569983 10.1167/14.2.17

[36] Jiang, A. Q. et al. Mistral 7b. arXiv preprint arXiv:2310.06825 (2023).

[37] Shepard, R. N. Geometrical approximations to the structure of musical pitch. *Psychol. Rev.* **89**, 305 (1982). 7134331 10.1037/0033-295X.89.4.305

[38] Jacoby, N. *et al.* Universal and non-universal features of musical pitch perception revealed by singing. *Curr. Biol.* **29**, 3229–3243 (2019). 31543451 10.1016/j.cub.2019.08.020 PMC9907018

[39] Anglada-Tort, M., Harrison, P. M., Lee, H. & Jacoby, N. Large-scale iterated singing experiments reveal oral transmission mechanisms underlying music evolution. *Curr. Biol.* **33**, 1472–1486 (2023). 36958332 10.1016/j.cub.2023.02.070

[40] Majid, A. *et al.* Differential coding of perception in the world’s languages. *Proc. Natl. Acad. Sci.* **115**, 11369–11376 (2018). 30397135 10.1073/pnas.1720419115 PMC6233065

[41] Blasi, D. E., Henrich, J., Adamou, E., Kemmerer, D. & Majid, A. Over-reliance on English hinders cognitive science. *Trends Cognit. Sci.* (2022). 10.1016/j.tics.2022.09.015 36253221

[42] Casasanto, D. Who’s afraid of the big bad Whorf? Crosslinguistic differences in temporal language and thought. *Lang. Learn.* **58**, 63–79 (2008). 10.1111/j.1467-9922.2008.00462.x

[43] Winawer, J. *et al.* Russian blues reveal effects of language on color discrimination. *Proc. Natl. Acad. Sci.* **104**, 7780–7785 (2007). 17470790 10.1073/pnas.0701644104 PMC1876524

[44] Abdou, M. et al. Can language models encode perceptual structure without grounding? A case study in color. arXiv preprint arXiv:2109.06129 (2021).

[45] Chaabouni, R., Kharitonov, E., Dupoux, E. & Baroni, M. Communicating artificial neural networks develop efficient color-naming systems. *Proc. Natl. Acad. Sci.* **118**, e2016569118 (2021). 33723064 10.1073/pnas.2016569118 PMC8000426

[46] Tucker, M., Levy, R., Shah, J. A. & Zaslavsky, N. Trading off utility, informativeness, and complexity in emergent communication. *Adv. Neural Inf. Process. Syst.* **35**, 22214–22228 (2022).

[47] Paramei, G. V., Griber, Y. A. & Mylonas, D. An online color naming experiment in Russian using Munsell color samples. *Color Res. Appl.* **43**, 358–374 (2018). 10.1002/col.22190

[48] Hebart, M. N., Zheng, C. Y., Pereira, F. & Baker, C. I. Revealing the multidimensional mental representations of natural objects underlying human similarity judgements. *Nat. Hum. Behav.* **4**, 1173–1185 (2020). 33046861 10.1038/s41562-020-00951-3 PMC7666026

[49] Zhuo, T. Y., Huang, Y., Chen, C. & Xing, Z. Exploring AI ethics of ChatGPT: A diagnostic analysis. arXiv preprint arXiv:2301.12867 (2023).

[50] Shepard, R. N. Toward a universal law of generalization for psychological science. *Science* **237**, 1317–1323 (1987). 3629243 10.1126/science.3629243

[51] Sims, C. R. Efficient coding explains the universal law of generalization in human perception. *Science* **360**, 652–656 (2018). 29748284 10.1126/science.aaq1118

[52] Marjieh, R., Griffiths, T. L. & Jacoby, N. Musical pitch has multiple psychological geometries. *bioRxiv*. 10.1101/2023.06.13.544763 (2023).

[53] Harrison, P. *et al.* Gibbs sampling with people. In Larochelle, H., Ranzato, M., Hadsell, R., Balcan, M. F. & Lin, H. (eds.) *Advances in Neural Information Processing Systems*. Vol. 33. 10659–10671 (Curran Associates, Inc., 2020).

[54] Woods, K. J., Siegel, M. H., Traer, J. & McDermott, J. H. Headphone screening to facilitate web-based auditory experiments. *Attent. Percept. Psychophys.* **79**, 2064–2072 (2017). 10.3758/s13414-017-1361-2 PMC5693749 28695541

[55] Brown, W. Some experimental results in the correlation of mental abilities 1. *Br. J. Psychol.* **1904–1920**(3), 296–322 (1910).

[56] Kriegeskorte, N., Mur, M. & Bandettini, P. A. Representational similarity analysis-connecting the branches of systems neuroscience. *Front. Syst. Neurosci.* **2**, 4 (2008). 19104670 10.3389/neuro.06.004.2008 PMC2605405

[57] Clark, J. The Ishihara test for color blindness. *Am. J. Physiol. Opt.* (1924).

[58] Rand, W. M. Objective criteria for the evaluation of clustering methods. *J. Am. Stat. Assoc.* **66**, 846–850 (1971). 10.1080/01621459.1971.10482356

