# Supplementary material for: Large language models predict human sensory judgments across six modalities
Source: Sci Rep. 2024 Sep 13;14:21445. doi: 10.1038/s41598-024-72071-1 (PMC11399123; doi:10.1038/s41598-024-72071-1)
Supplement: Supplementary file 1 — Supplementary Information. [file 41598_2024_72071_MOESM1_ESM.pdf]

Large Language Models Predict Human Sensory Judgments Across Six Modalities

Supplementary Information

Raja Marjieh[1,*], Ilia Sucholutsky[2], Pol van Rijn[3], Nori Jacoby[3,4,†], and Thomas L. Griffiths[1,2,†]

[1]Department of Psychology, Princeton University, USA
[2]Department of Computer Science, Princeton University, USA
[3]Max Planck Institute for Empirical Aesthetics, Germany
[4]Department of Psychology, Cornell University, USA
[*]raja.marjieh@princeton.edu
[†]Equal contribution.

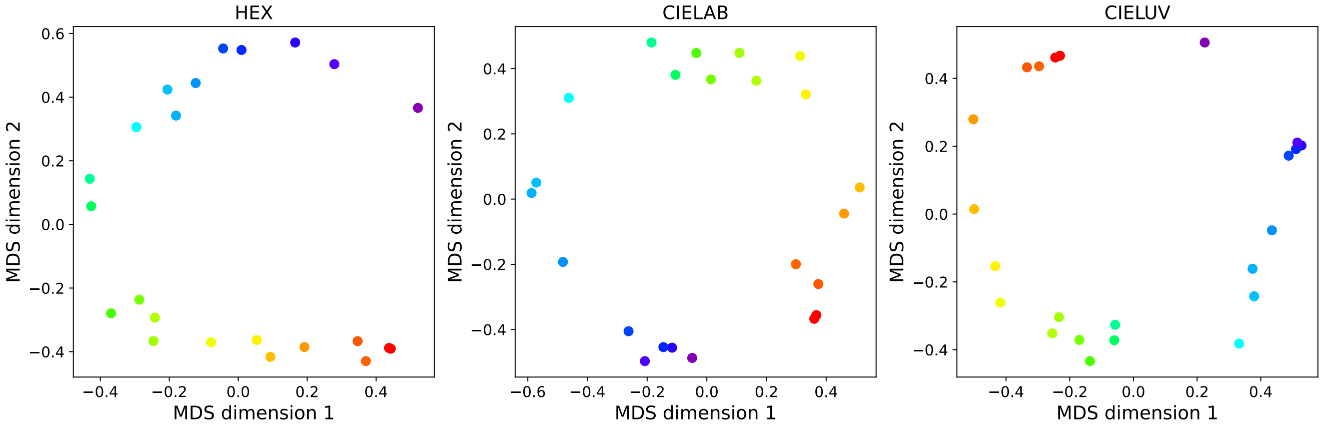

Figure S1. Multidimensional scaling solutions for GPT-4 similarity judgments elicited using different color notations and in zero-shot fashion (i.e. without any prompt examples).

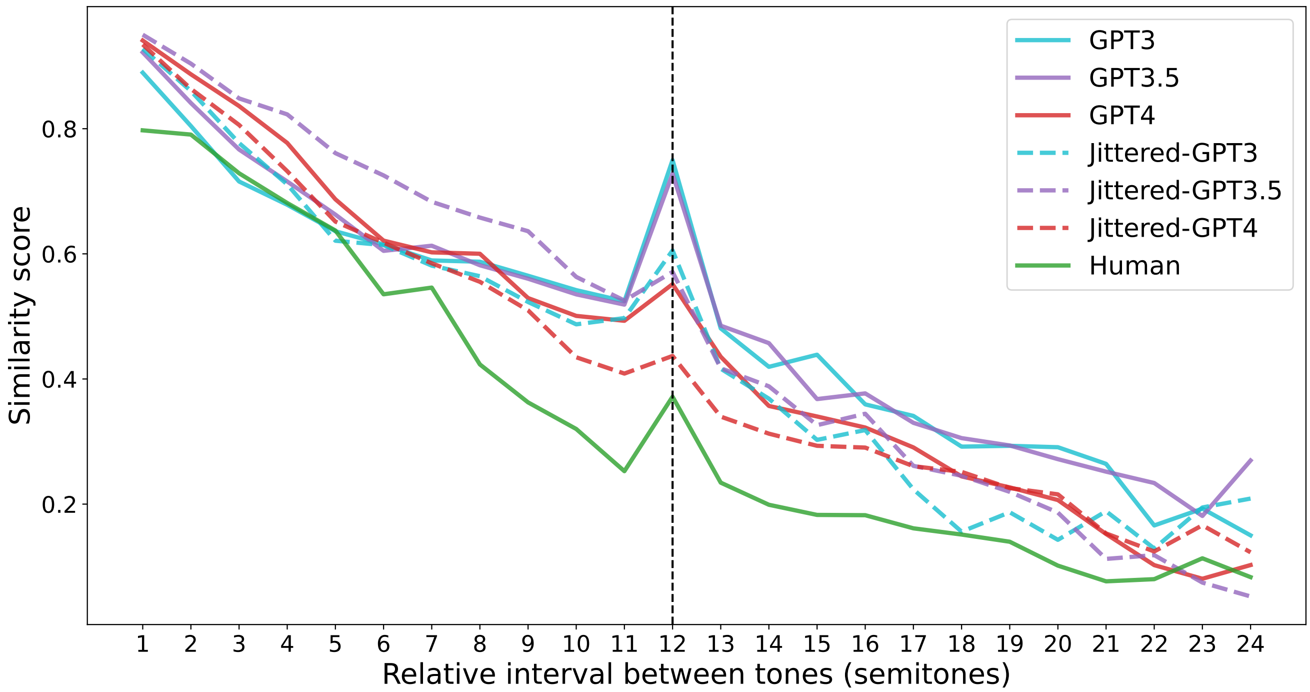

Figure S2. Average similarity scores as a function of the relative interval between tones for original (solid) and jittered pitch values (dashed).

Explicit GPT prompts

GPT prompt elicitation experiments were conducted using the OpenAI Text Completion (for GPT-3) and Chat Completion (for GPT-3.5 and GPT-4) APIs. For all experiments with the Text API model, we used the last available version of GPT-3 on that API (i.e., text-davinci-003). For the experiments in the main text involving the Chat API models we used the versions snapshotted in March 2023 (i.e., gpt-4-0314, gpt-3.5-turbo-0301). However, support for those snapshots was recently dropped and our control experiments (jittered pitch similarity, color tags similarity, and color name similarity) were run using the June 2023 snapshots (i.e., gpt-4-0613, gpt-3.5-turbo-0613). For the experiment involving GPT-4V, we used the preview version (i.e., gpt-4-vision-preview) which is the only one available as of the time of writing. The temperature was set to 0.7 for similarity judgments and 0 for color naming experiments and GPT-4V similarity judgment experiments. The exact prompt formats used are shown below.

Similarity judgments

Color:

```
People described pairs of colors using their hex codes.  How similar are the
two colors in each pair on a scale of 0-1 where 0 is completely dissimilar and
1 is completely similar?  Respond only with the numerical similarity rating.
Color 1:  #ff5700 Color 2:  #ff9b00 Rating 0.76
Color 1:  #b3ff00 Color 2:  #00ff61 Rating:  0.45
Color 1:  #FF0000 Color 2:  #00b2ff Rating:  0.02
Color 1:  <hex-code1> Color 2:  <hex-code2>
Rating:
```

Pitch:

```
People described pairs of musical notes using their frequencies in hertz.
How similar are the musical notes in each pair on a scale of 0-1 where 0 is completely
dissimilar and 1 is completely similar?

Note 1:  587.3295358348151 Hz
Note 2:  987.7666025122483 Hz
Rating:  0.46083740655517463

Note 1:  349.2282314330039 Hz
Note 2:  277.1826309768721 Hz
Rating:  0.743838237117938

Note 1:  415.3046975799451 Hz
Note 2:  987.7666025122483 Hz
Rating:  0.19874605585261726

Note 1:  <frequency1>
Note 2:  <frequency2>
Rating:
```

Vocal consonants:

```
People described vocal consonants using the international phonetic alphabet (IPA).
How similar do the vocal consonants in each pair sound on a scale of 0-1 where
0 is completely dissimilar and 1 is completely similar?
Respond only with the numerical similarity rating.

Vocal Consonant 1:  f
Vocal Consonant 2:  m
Rating:  0.5

Vocal Consonant 1:  n
Vocal Consonant 2:  ʒ
Rating:  0.40740740740740744

Vocal Consonant 1:ʃ
Vocal Consonant 2:ʃ
Rating:  1.0

Vocal Consonant 1:  <consonant1>
Vocal Consonant 2:  <consonant2>
Rating:
```

Loudness:

```
People described the loudness of pure tones in decibels (dB).
How similar do the pure tones in each pair sound on a scale of 0-1 where 0 is
completely dissimilar and 1 is completely similar?

Pure Tone 1:  72.6 dB
Pure Tone 2:  74.1 dB
Rating:  0.3495324720283043

Pure Tone 1:  74.6 dB
Pure Tone 2:  73.6 dB
Rating:  0.5055839477695901

Pure Tone 1:  74.1 dB
Pure Tone 2:  74.1 dB
Rating:  1.0

Pure Tone 1:  <loudness1>
Pure Tone 2:  <loudness2>
Rating:
```

Taste:

```
People described flavors they tasted using words.
How similar are the flavors in each pair on a scale of 0-1 where 0 is completely
dissimilar and 1 is completely similar?

Flavor 1:  quinine
Flavor 2:  artificial sweetener
Rating:  0.0

Flavor 1:  artificial sweetener
Flavor 2:  salt
Rating:  0.015433904145892428

Flavor 1:  quinine-sugar
Flavor 2:  acid-sugar
Rating:  0.2539115246067999

Flavor 1:  <flavor1>
Flavor 2:  <flavor2>
Rating:
```

Timbre:

```
People listened to pairs of musical instruments and rated the similarity of their
timbre.
How similar is the timbre of the instruments in each pair on a scale of 0-1 where
0 is completely dissimilar and 1 is completely similar?

Instrument 1:  Cello
Instrument 2:  Flute
Rating:  0.5604846433040316

Instrument 1:  Flute
Instrument 2:  Clarinet
```

```
Rating:  0.270932601836378

Instrument 1:  Trombone
Instrument 2:  Bassoon
Rating:  0.2893895067551666

Instrument 1:  <instrument1>
Instrument 2:  <instrument2>
Rating:
```

STEP-Tag word associations control

```
People described pairs of colors using tags.  How similar are the two colors
in each pair on a scale of 0-1 where 0 is completely dissimilar and 1
is completely similar?  Respond only with the numerical similarity rating.

Color 1:  autumn, colour of an orange, fire, warm, warmth
Color 2:  autum, golden, orange, sunset
Rating:  0.76

Color 1:  bright, colour of grass, light green, luminous, neon, vibrant
Color 2:  bright, fresh, vibrant
Rating:  0.45

Color 1:  alert, blood, bold, bright, danger, red, vibrant, warning
Color 2:  bright, calm, cool, light, sky, water
Rating:  0.02

Color 1:  <color1>
Color 2:  <color2>
Rating:
```

Color terms control

```
People described pairs of colors using their names.  How similar are the two
colors in each pair on a scale of 0-1 where 0 is completely dissimilar and 1
is completely similar?  Respond only with the numerical similarity rating.

Color 1:  orange (pantone)
Color 2:  vivid gamboge
Rating 0.76

Color 1:  bitter lime
Color 2:  guppie green
Rating:  0.45

Color 1:  red
Color 2:  blue bolt
Rating:  0.02

Color 1:  <color-name1>
Color 2:  <color-name2>
Rating:
```

Color naming

Basic color free-elicitation:

English:

Name 15 basic colors.

Russian:

Перечислите 15 основных цветов.

Color naming elicitation:
English:

Here is a list of 15 basic color names: <shuffled basic color list>.
Which of these names best describes the following color: <hex-code>?
Respond only using the name.

Russian:

Вот список из 15 названий основных цветов: <shuffled basic color list> .
Какое из названий цветов лучше всего описывает следующий цвет: <hex-code>?
Отвечайте только названием одного цвета из списка.

Russian translation of the prompt was checked by author IS who is a native speaker. We repeated this prompt ten times for each WCS color with the basic color list shuffled each time and temperature set to the default 0.7 to elicit ten names per WCS color. For each of the ten elicitations per color, if the output was not one of the 15 basic colors we would keep re-querying GPT until it did give a valid color output (GPT-4 is slightly non-deterministic even at temperature 0 due to changes in hardware). If after 10 attempts the response was still invalid, we would return "error" as the color (this response is later discarded from the analysis).

Additional controls for in-context example prompting

The goal of the provided prompt examples was to ensure that the LLMs return numerical predictions. However, this is really necessary only for GPT-3 and so to avoid any concerns regarding reliance on those examples we ran a zero-shot (no example) control analysis for GPT-3.5 and 4 as these allow for such an elicitation. Overall, the predictive power of the models persisted: for GPT-4 without any examples, the human-model correlations were: .85 (CI $[.84, .85]$) for color, .77 (CI $[.75, .79]$) for pitch, .69 (CI $[.50, .89]$) for loudness, .60 (CI $[.59, .61]$) for taste, .59 (CI $[.58, .60]$) for consonants, and .36 (CI $[.34, .38]$) for timbre. Likewise, for GPT-3.5 we have: .82 (CI $[.80, .84]$) for color, .81 (CI $[.79, .83]$) for pitch, .40 (CI $[.27, .54]$) for loudness, .57 (CI $[.55, .59]$) for consonants, .52 (CI $[.49, .56]$) for taste, and .26 (CI $[.24, .28]$) for timbre. This suggests that the models' ability to predict human sensory judgments is not critically contingent on the prompting-with-examples approach, but merely enhanced by it.

Additional controls for color notation

To check whether the results were dependent on the choice of color notation, we repeated the GPT-4 similarity experiments using CIELAB, CIELUV as well as hex-code color notations without any prompt examples. We converted the hex codes to corresponding CIELAB and CIELUV values using the Python library `scikit-image`. Then, we computed the model-human correlations as before as well as the corresponding MDS solutions. We found that in all cases the correlations were high with 95% CIs given by $[.84, .85]$ for hex-code, $[.78, .80]$ for CIELAB, and $[.83, .85]$ for CIELUV. Likewise, all conditions reproduced the color wheel as can be seen in Supplementary Figure S1. These results suggest that the model is robust to these transformations in the similarity domain.

As for the color naming domain, we repeated the English naming study in CIELAB and CIELUV coordinates with GPT-4. Unlike the similarity domain, we found that GPT-4's performance was quite impacted by these transformations, yielding much lower human-model ARI metrics ($ARI = 0.19$, 95% CI $[0.18, 0.21]$ for CIELAB, and $ARI = 0.21$, 95% CI $[0.19, 0.23]$ for CIELUV). These results suggest that the LLM did not know to perform the conversion in this context. While this could potentially be improved by explicitly instructing the model in the prompt to do the conversion prior to naming, it sheds light on the possibility that the source of information that the model relies on to perform the naming task is much more heavily based on hex-code data as these are very common in HTML documents that are part of the model's training set.

Experiments with an open-source LLM

To improve the reproducibility of our work, we include additional experiments using an open-source LLM known as Mistral-7B (Version Mistral-7B-Instruct-v0.3[1]) for both the similarity and color naming domains. In the similarity domain, we found that the model was able to yield reliable predictions of human judgments with the human-model correlation 95% CIs being:

[1] https://huggingface.co/mistralai/Mistral-7B-Instruct-v0.3

$[.74,.77]$ for color, $[.66,.77]$ for loudness, $[.79,.81]$ for pitch, $[.41,.45]$ for taste, $[.31,.46]$ for timbre, and $[.39,.45]$ for vocal consonants. As for the color naming domain, we ran the English color naming experiment (as the model was trained on English text; Supplementary Figure S4 and found that the model also yielded a significant human-model ARI of .31 (95% CI [.28,.33]). However, this value is lower than that of the GPT variants which is not surprising given that Mistral-7B is a much smaller model.

Experiments with embedding models

To further test whether our similarity predictions can be derived from simpler embedding models, we further considered two standard models: GloVe[2], and ADA[3]. Starting from GloVe, we found that most of the compound labels necessary for capturing the nuanced differences in stimulus specifications were simply not part of the model's dictionary. Timbre was the only exception, however, the model yielded practically no correlations with human data ($r = .035$). As for ADA, in this case we could embed all stimuli as compound sentences that indicate the domain name and the stimulus specification (e.g., 'Color: #2800ff'). In this case, the resulting correlations were: $r = .03$ for timbre, $r = .56$ for loudness, $r = .28$ for color, $r = .12$ for taste, $r = .24$ for pitch, and $r = .22$ for vocal consonants. We see that these results are non-zero (with the exception of timbre which again was challenging for the embedding model) but still much lower than those derived from the LLMs, with the exception of loudness which yielded intermediate correlations. These results further suggest that capturing nuanced sensory judgments is not straightforward using standard embedding models.

[2] https://github.com/stanfordnlp/glove

[3] https://openai.com/blog/new-and-improved-embedding-model

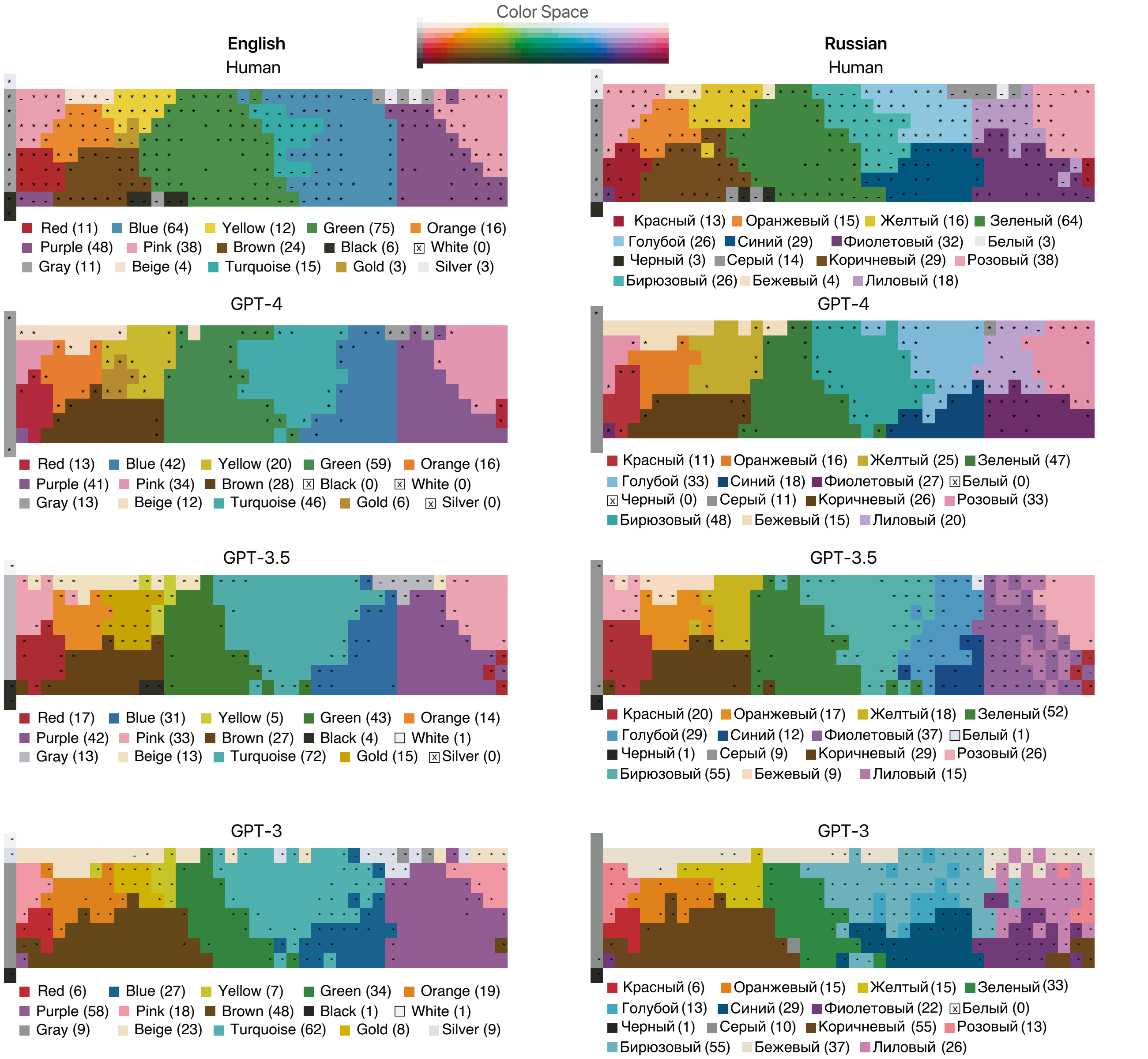

Figure S3. Color naming experiment using 330 Munsell colors from the World Color Survey. Data comparison between humans and LLMs in Russian and English. Participants and LLMs were shown colors and were asked to choose from the same 15-color list. The count of chosen colors for each option is given in parentheses. The color of a response cluster in the maps represents its average color.

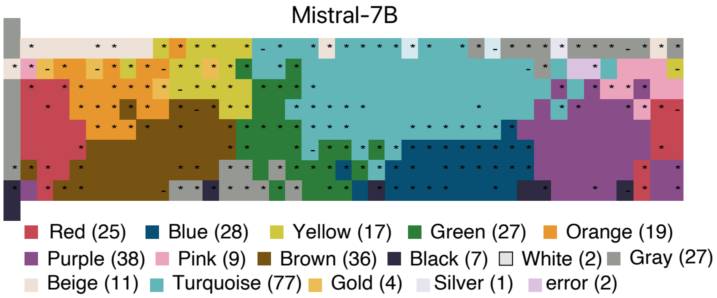

Figure S4. Color naming experiment in English using 330 Munsell colors from the World Color Survey for Mistral-7B. For two color chips the model did not follow the instructions in which case it was indicated by an “error” label.
